# Climate Change and New Challenges for Rural Communities: Particulate Matter Matters

**DOI:** 10.3390/su152316192

**Published:** 2023-11-22

**Authors:** Isabelle Racine Miousse, Rachel B. Hale, Scott Alsbrook, Gunnar Boysen, Tanya Broadnax, Carleisha Murry, Candace Williams, Chul Hyun Park, Robert Richards, Justin Reedy, Marie-Cécile Chalbot, Ilias G. Kavouras, Igor Koturbash

**Affiliations:** 1 Department of Environmental Health Sciences, Fay W. Boozman College of Public Health, University of Arkansas for Medical Sciences, Little Rock, AR 72205, USA; 2 Department of Biochemistry and Molecular Biology, College of Medicine, University of Arkansas for Medical Sciences, Little Rock, AR 72205, USA; 3 Rural Community Alliance, Little Rock, AR 72205, USA; 4 Clinton School of Public Service, University of Arkansas, Little Rock, AR 72201, USA; 5 Department of Communication, University of Oklahoma, Norman, OK 73019, USA; 6 Department of Biological Sciences, New York City College of Technology, City University of New York, New York, NY 10018, USA; 7 Department of Environmental, Occupational and Geospatial Health Sciences, City University of New York, New York, NY 10018, USA

**Keywords:** agriculture, citizen science, climate change, geographic information systems, inhalational toxicology, DNA methylation, particulate matter, rural communities, wildfires

## Abstract

Climate change presents multiple challenges to rural communities. Here, we investigated the toxicological potential of the six types of particulate matter most common to rural Arkansas: soil, road, and agricultural dusts, pollen, traffic exhaust, and particles from biomass burning in human small airway epithelial cells (SAECs). Biomass burning and agricultural dust demonstrated the most potent toxicological responses, exhibited as significant (*p* < 0.05) up-regulation of *HMOX1* (oxidative stress) and *TNFα* (inflammatory response) genes as well as epigenetic alterations (altered expression of DNA methyltransferases *DNMT1*, *DNMT3A*, and *DNMT3B*, enzymatic activity, and DNA methylation of alpha satellite elements) that were evident at both 24 h and 72 h of exposure. We further demonstrate evidence of aridification in the state of Arkansas and the presence of winds capable of transporting agricultural dust- and biomass burning-associated particles far beyond their origination. Partnerships in the form of citizen science projects may provide important solutions to prevent and mitigate the negative effects of the rapidly evolving climate and improve the well-being of rural communities. Furthermore, the identification of the most toxic types of particulate matter could inform local policies related to agriculture, biomass burning, and dust control.

## 1. Introduction

Climate change presents multiple health challenges for rural residents in the United States (US). Rural communities are highly vulnerable to health impacts from climate change due to limited adaptive capacity, existing health disparities, and high-exposure occupations [1]. Increasing wildfires and droughts due to climate change place a significant burden on the already poor respiratory health of rural residents, especially those located along the southern half of the Mississippi River [2,3].

Intensifying and severe high and low precipitation events associated with climate change are of particular concern as the effects can be multifaceted. Alongside direct effects such as injury and death, indirect effects, such as increasing exposure to particulate matter, can lead to serious long-term health consequences. For instance, wildfires have been traditionally localized to California, the southwestern US, and British Columbia. Due in part to climate change, wildfires have expanded their range to impact the northeastern US and have occurred in the tropical humid climate found in the southeastern US. The year 2023 has seen a number of large-scale wildfires, involving new locations, such as Maui and eastern Canada. The frequency and intensity of wildfires have increased in the last decade and are predicted to further increase by at least 30% by 2050 [4,5]. Importantly, the effects of larger wildfires can expand well beyond the affected areas. In 2023, Canadian wildfires resulted in a significant increase in asthma syndrome emergency room visits in New York City, located hundreds of miles away [6]. As a result, the number of communities directly and indirectly affected by wildfires will continue to grow.

Another dangerous consequence associated with drought is the wider and larger amplitude of the spread of chemical residues from bare farmlands or exposed sediments from desiccating bodies of water. The best-known example of this is the Aral Sea—formerly the fourth-largest lake in the world, located between Kazakhstan and Uzbekistan. During the Soviet Union era, the lake and its basin were extensively used not only for its rich fish resources but also for agriculture, mostly cotton production [7]. Two rivers that fed this lake, Amu Darya and Syr Darya, were unsustainably used for irrigation of large cotton fields, which substantially reduced the water supply to the lake. Due to its location in the desert, the lake suffered significant losses of water because of high evaporation rates. Furthermore, runoffs from the fields were directed back into the rivers, resulting in the accumulation of agrochemicals in the lake for several decades [7,8]. As a consequence, today the lake is nearly nonexistent, which has irreversibly affected local economies. Furthermore, frequent desert winds stimulated the drift of agrochemicals from the exposed lakebed into the neighboring communities, causing increases in respiratory and other diseases, including cancer incidence [9,10]. In the US, a number of large lakes, i.e., Lake Mead in Nevada and Salton Sea in California, now resemble the features of the Aral Sea catastrophe [11–13].

Arkansas (AR), an agricultural state with 41% of the state’s population living in rural counties [14], is not an exception to global trends in climate change and experiences its own challenges. In the last decade, Arkansas has begun to experience severe high and low precipitation events which have resulted in severe drought impacting rural counties in eastern Arkansas. Of particular concern are the Delta areas along the Mississippi River, the vast majority of which are farmlands. Drought, aridification, and winds increase the drift of agricultural residues into the neighboring communities (consisting mostly of underserved and underrepresented populations), which then increases both inhalational and ingestion exposure of these residues. These exposures may lead to increased rates of respiratory and other disease. This is especially true for a number of low-socioeconomic rural Arkansas counties along the Mississippi Delta, such as Chicot, Lee, and Philips counties [15].

We have demonstrated previously that particulate matter (PM) characteristic of the state of Arkansas, besides displaying strong genotoxic potential, also exhibits epigenotoxicity (that is, not associated with damage to DNA, but rather affecting DNA modifications, i.e., DNA methylation) [16]. Furthermore, we have demonstrated that samples of agricultural dust (AD) collected from farmlands in rural Arkansas induced the highest degree of biochemical and molecular alterations in macrophages compared with soil dust (SD), road dust (RD), traffic exhaust (TE), biomass burning (BB), and pollen [17].

While southeast Arkansas mainly comprises farmlands, central and northwest areas of the state are predominantly occupied by deciduous forests. Furthermore, recent years have been characterized by extensive wildfires in neighboring Louisiana [18,19]. These events will exacerbate the existing health disparities felt by many Arkansas communities due to low socioeconomic status, race, limited access to quality healthcare services, and rurality.

Due in part to polarization and misinformation, Arkansans are less likely to believe in climate change or how it may impact their physical and mental health compared with other US states [20]. Rural communities in the Arkansas Delta have expressed concerns about air quality and associated respiratory health issues [21]. Residents of these communities face challenges in addressing these concerns due to limited adaptive capacity.

In this article, we investigated the toxicological profile of six sources of dust particles that are common to the state of Arkansas. We further provide evidence of the magnitude of negative effects caused by exposures to those sources (AD and BB) using geospatial approaches and discuss potential mitigation and prevention strategies and the challenges of their administration in rural communities.

## 2. Materials and Methods

### 2.1. Dust Sample Collection, Preparation, and Characterization

Samples that were collected in the spring of 2014 and used in our previous study [17] were utilized here. The following samples were collected: soil dust (SD), road dust (RD), agricultural dust (AD), traffic exhaust (TE), biomass burning (BB), and pollen (P). Briefly, SD was collected at the state park; RD was collected at the curbside of the residential street in Little Rock, AR, USA. Samples of AD were derived from farmlands in rural Arkansas. Pollen was collected from the state park. Aerosol samples—TE and BB particles—were collected on quartz filters. For this purpose, a PM_10_ Harvard Impactor (HI) sampler (Air Diagnostics and Engineering, Inc., Harrison, ME, USA) was utilized [22]. TE was collected at the parking garage at UAMS (Little Rock, AR, USA) from 6 a.m. to 6 p.m. (Monday—Friday) on a 20.3 cm × 25.4 cm pre-fired quartz filter mounted on a high-volume sampler (Tisch Environmental, Cleves, OH, USA). BB samples were collected by burning the commercially available wood logs in a fireplace [23]. Particle preparation and morphological and chemical characterization were reported elsewhere [17].

### 2.2. Cell Culture and Exposure

Human small alveolar epithelial cells (SAECs) were purchased from the American Type Culture Collection. Cells were grown and maintained according to the manufacturer’s protocol (ATCC, Rockville, MD, USA). Cells were exposed to either 0, 5, or 50 μg/mL of water-soluble extracts of the abovementioned PM samples. The doses were calculated based on previous studies [24]. Cells were trypsinized after 24 h or 72 h of exposure, flash-frozen in liquid nitrogen, and stored at −80 °C until further analysis.

### 2.3. DNA and RNA Extraction

DNA and RNA were extracted using the AllPrep Mini Kit (Qiagen, Valencia, CA, USA), as previously described [17].

### 2.4. Gene Expression Analysis

Complementary DNA (cDNA) was synthesized from 1 μg RNA using random primers and a High-Capacity cDNA Reverse Transcription Kit (Applied Biosystems, Foster City, CA, USA). Quantitative real-time PCR (qRT-PCR) was performed to determine the levels of gene transcripts [16,17,25].

### 2.5. DNA Methylation Analysis

DNA methylation of retrotransposons LINE-1 and Alu was assessed via methylation-sensitive qRT-PCR as previously described [16,17,26].

### 2.6. Analysis of the Methylation Status of Satellite DNA

DNA methylation of alpha satellite repetitive elements was assessed via methylation-sensitive McrBC-qRT-PCR [27,28].

### 2.7. Methyltransferase Activity

The methyltransferase activity of the nuclear extracts was analyzed via fluorometry using the EpiQuik DNA Methyltransferase (DNMT) Activity/Inhibition Assay Kit (Epigentek, Farmingdale, NY, USA) according to the manufacturer’s protocol.

### 2.8. Geospatial Analysis

Data from the US Drought Monitoring Project (USDM) were used to calculate a Drought Severity and Coverage Index (DSCI) for 100-square-kilometer hexagonal tessellations and averaged for the 21 September 2022 to 1 November 2022 timeframe [29]. Additionally, data from regional weather stations and inverse distance weighting (IDW) were used to interpolate a raster surface for average wind speed for the same timeframe [30]. These data were processed using ArcGIS Pro and Python in Jupyter Notebooks.

### 2.9. Statistical Analysis

One-way ANOVA, followed by Dunnett’s test, was used to assess the statistical significance between the treatment groups (GraphPad Prism 9.5.1, GraphPad Software, Boston, MA, USA). *p*-values ≤ 0.05 were considered as statistically significant.

## 3. Results

### 3.1. Molecular Effects of Exposure to Dust: Oxidative Stress, Inflammation, and Epigenetic Alterations

#### 3.1.1. Selective Effects of PM Exposure on Oxidative Stress and Inflammation Markers in SAEC Cells

**Figure 1.**
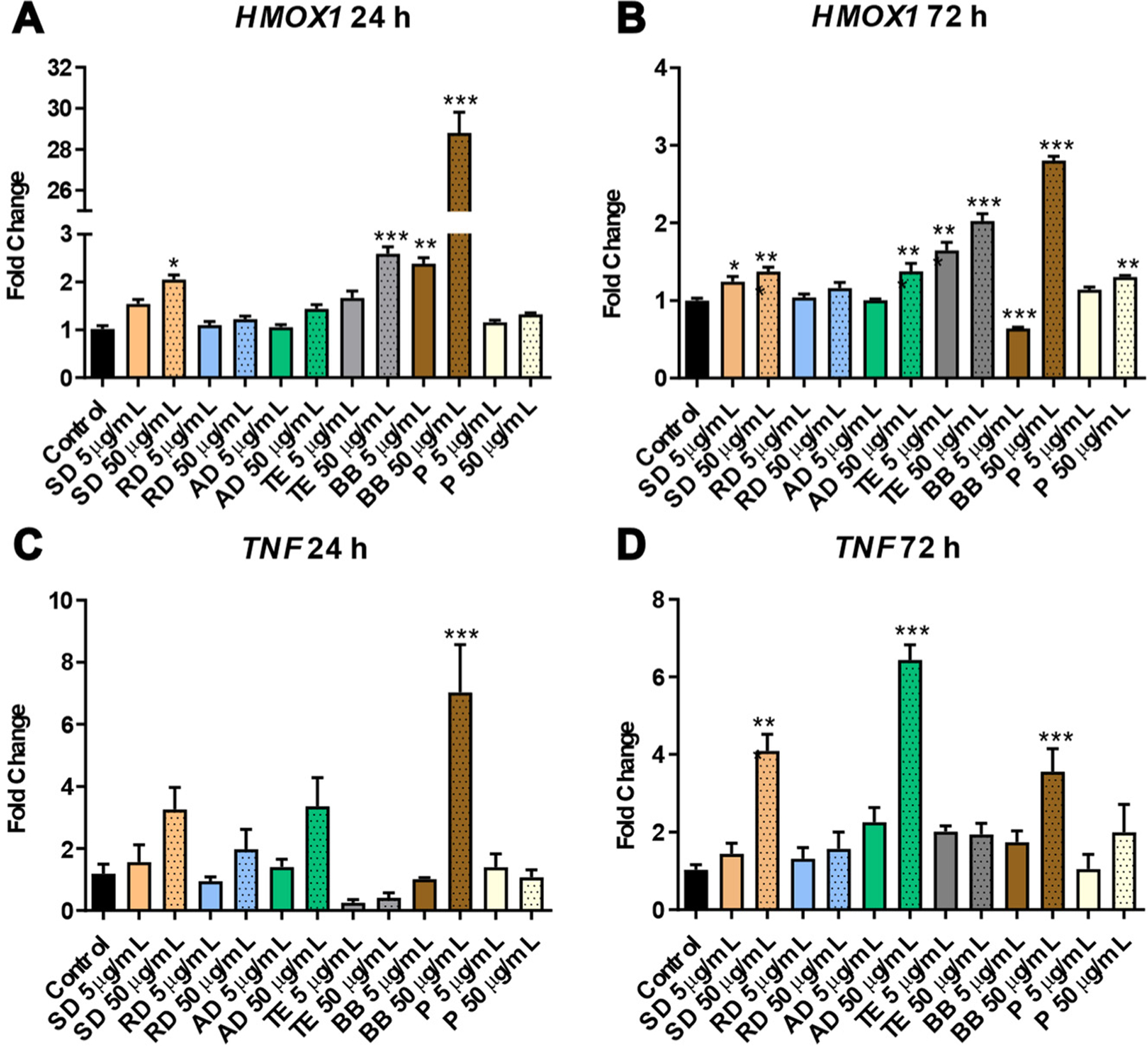
The differential expression of *Hmox1* (panels (**A**) and (**B**)) and *TNFα* (panels (**C**) and (**D**)) in human small alveolar epithelial cells (SAEC) after 24 and 72 h of exposure to a water-soluble fraction of six sources of particulate matter. Asterisks: (*) denotes significant (*p* < 0.05), (**) denotes significant (*p* < 0.01), and (***) denotes significant (*p* < 0.001) difference from control.

After 24 h of exposure, significant elevation of the *HMOX1* gene (a marker of oxidative damage to DNA) was observed in the 50 μg/mL SD and TE groups (2.1-fold, *p* < 0.05 and 2.6-fold, *p* < 0.001, respectively) and both doses of BB (2.5-fold, *p* < 0.01 and 28.8-fold, *p* < 0.01 for 5 and 50 μg/mL doses, respectively) (Figure 1A). At a 72 h time-point, a subtle but significant elevation of *HMOX1* was observed in response to SD exposure (1.3-fold, *p* < 0.05 and 1.4-fold, *p* < 0.001 for 5 and 50 μg/mL doses, respectively); to 50 μg/mL dose for AD and pollen (1.4-fold, *p* < 0.001 and 1.3-fold, *p* < 0.01, respectively); and after both doses of TE (1.7-fold, *p* < 0.001 and 2.0-fold, *p* < 0.001, respectively). Interestingly, the 5 μg/mL dose of BB resulted in a paradoxical decrease in *HMOX1* levels (0.6-fold, *p* < 0.001); at the dose of 50 μg/mL, however, *HMOX1* exhibited a significant increase in mRNA levels, although to a lower extent than at 24 h (2.8-fold, *p* < 0.001) (Figure 1B).

Insignificant increases in *TNFa* (a marker of inflammation) were observed after exposure to a 50 μg/mL dose of SD, RD, and AD (3.3-, 2.0-, and 3.4-fold, respectively).

A significant increase (7.0-fold, *p* < 0.001) was observed after exposure to a 50 μg/mL dose of BB samples (Figure 1C). At 72 h, exposure to 50 μg/mL doses of SD, AD, and BB resulted in significant increases in *TNFα* (4.1-, 6.4, and 3.6-fold, respectively, *p* < 0.001 for all) (Figure 1D).

In summary, exposure to several sources of PM collected in Arkansas exhibited potent pro-oxidative and pro-inflammatory responses, with the most pronounced effects observed in the case of AD and BB.

#### 3.1.2. Involvement of Epigenetic Mechanisms in Response to Exposure to PM

**Figure 2.**
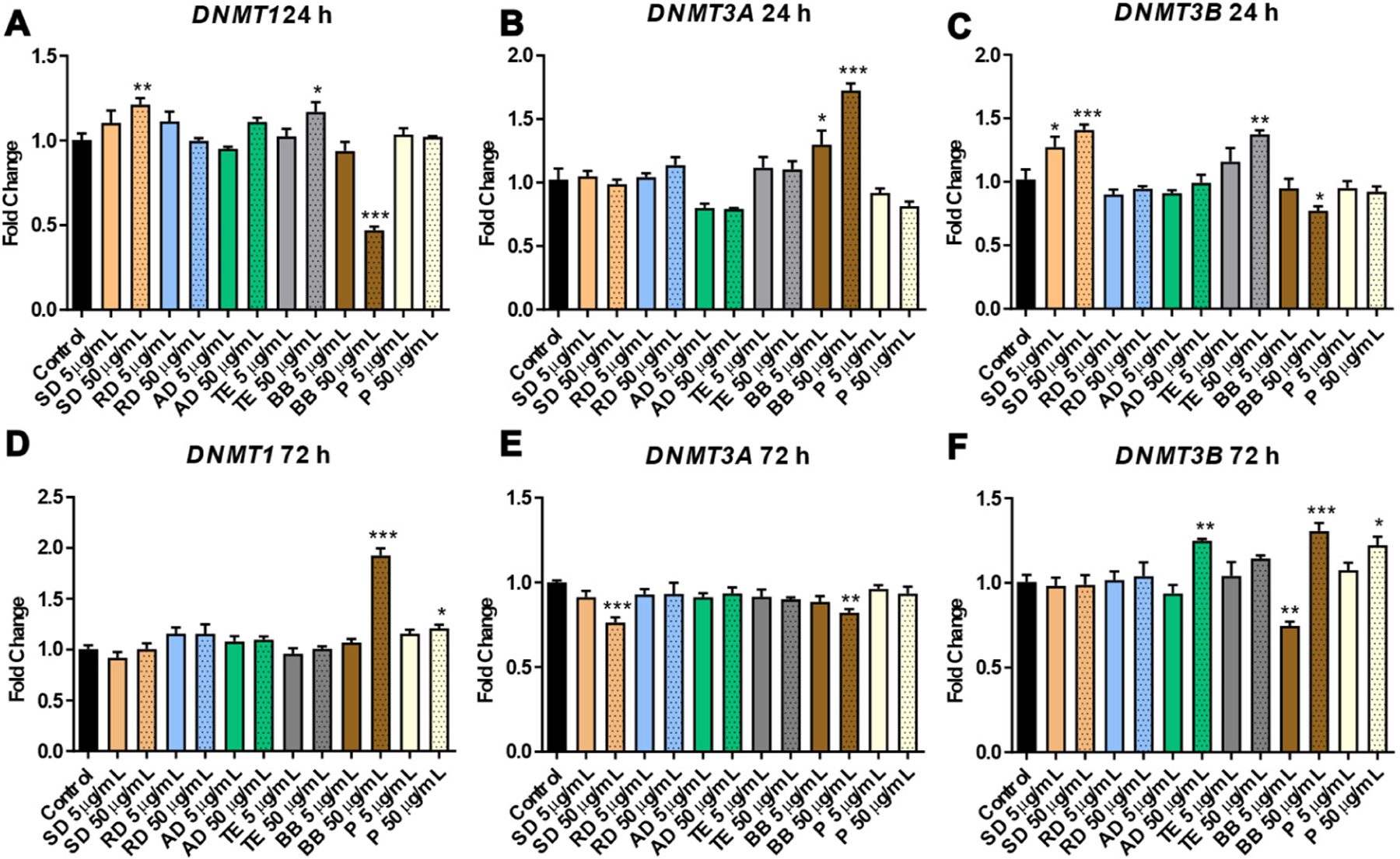
The differential expression of *Dnmt1*, *Dnmt3a*, and *Dnmt3b* at 24 h (h) (panels (**A**–**C**)) and 72 h (panels (**D**–**F**)) in human small alveolar epithelial cells (SAECs) after exposure to a water-soluble fraction of six sources of particulate matter. Asterisks: (*) denotes significant (*p* < 0.05), (**) denotes significant (*p* < 0.01), and (***) denotes significant (*p* < 0.001) difference from control.

**Figure 3.**
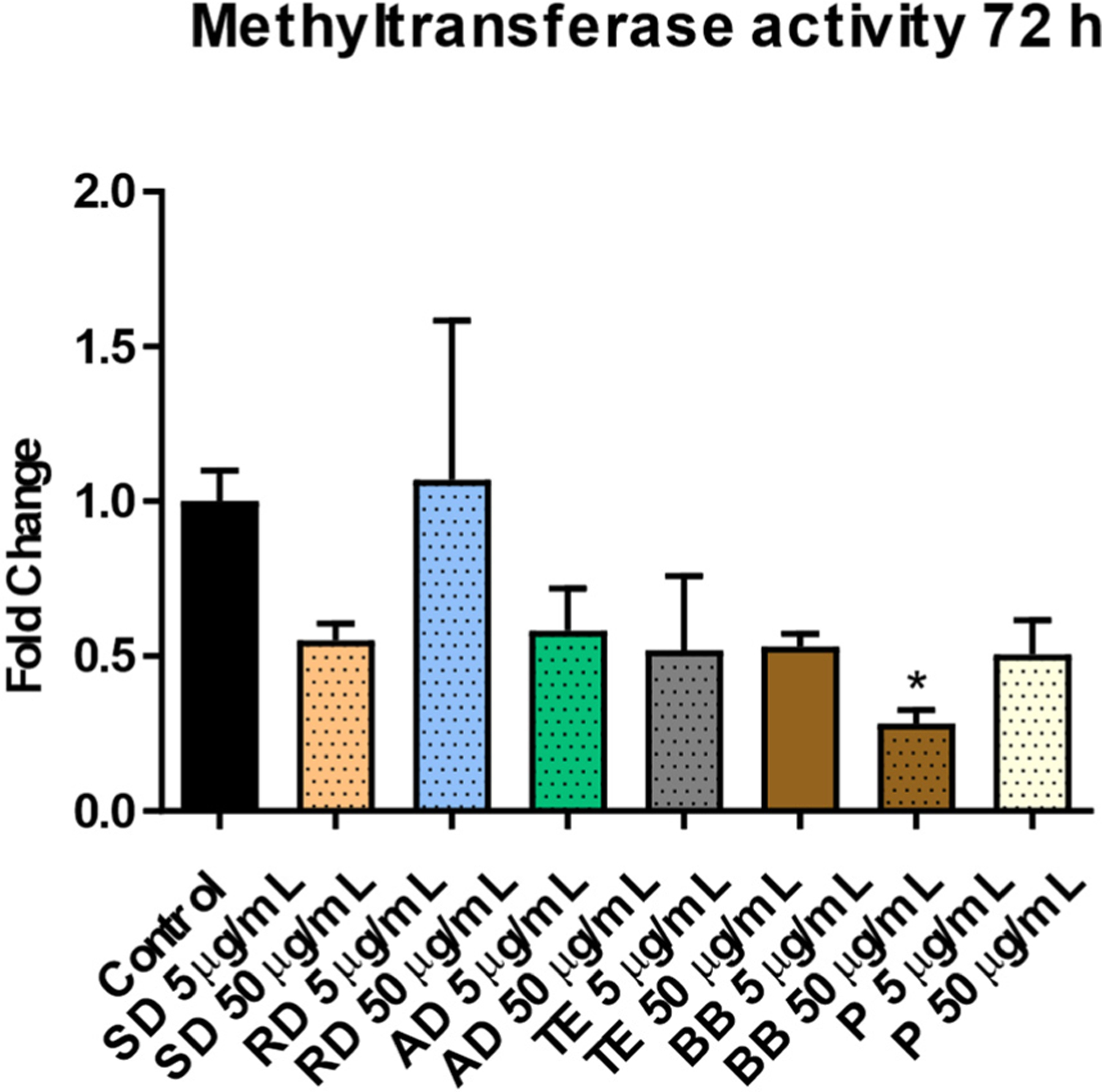
Nuclear DNA methyltransferase enzymatic activity after 72 h of exposure to a water-soluble fraction of six sources of particulate matter. Asterisk (*) denotes a significant (*p* < 0.05) difference from the control.

Dosing SAEC cells resulted in dynamic alterations in the expression of DNA methyltransferases—enzymes involved in either maintenance (*DNMT1*) or de novo (*DNMT3A* and *DNMT3B*) methylation. The highest extent of alterations was observed in response to exposure to BB, which resulted in significantly decreased levels of *DNMT1* and *DNMT3B* at 24 h (0.5-fold, *p* < 0.001 and 0.8-fold, *p* < 0.05, respectively), and opposite to this reactivation of both methyltransferases at 72 h (2.0-fold and 1.3-fold for *DNMT1* and *DNMT3B*, respectively; *p* < 0.001 for both) (Figure 2). Contrarily, increased mRNA levels of *DNMT3A* in response to BB were observed at an earlier time-point (1.3- and 1.7-fold, *p* < 0.05 and *p* < 0.001 for 5 μg/mL and 50 μg/mL doses, respectively) and a subtle but significant decrease was observed at 72 h (0.9-fold, *p* < 0.01). The altered expression of DNA methylation machinery was associated with the reduced methyltransferase activity that was observed after exposure to the 50 μg/mL dose of BB (0.3-fold, *p* < 0.05) (Figure 3). Exposure to other sources resulted in either insignificant or low-scale alterations in the expression of DNA methyltransferases, with most effects observed within the SD and AD groups (Figure 2).

**Figure 4.**
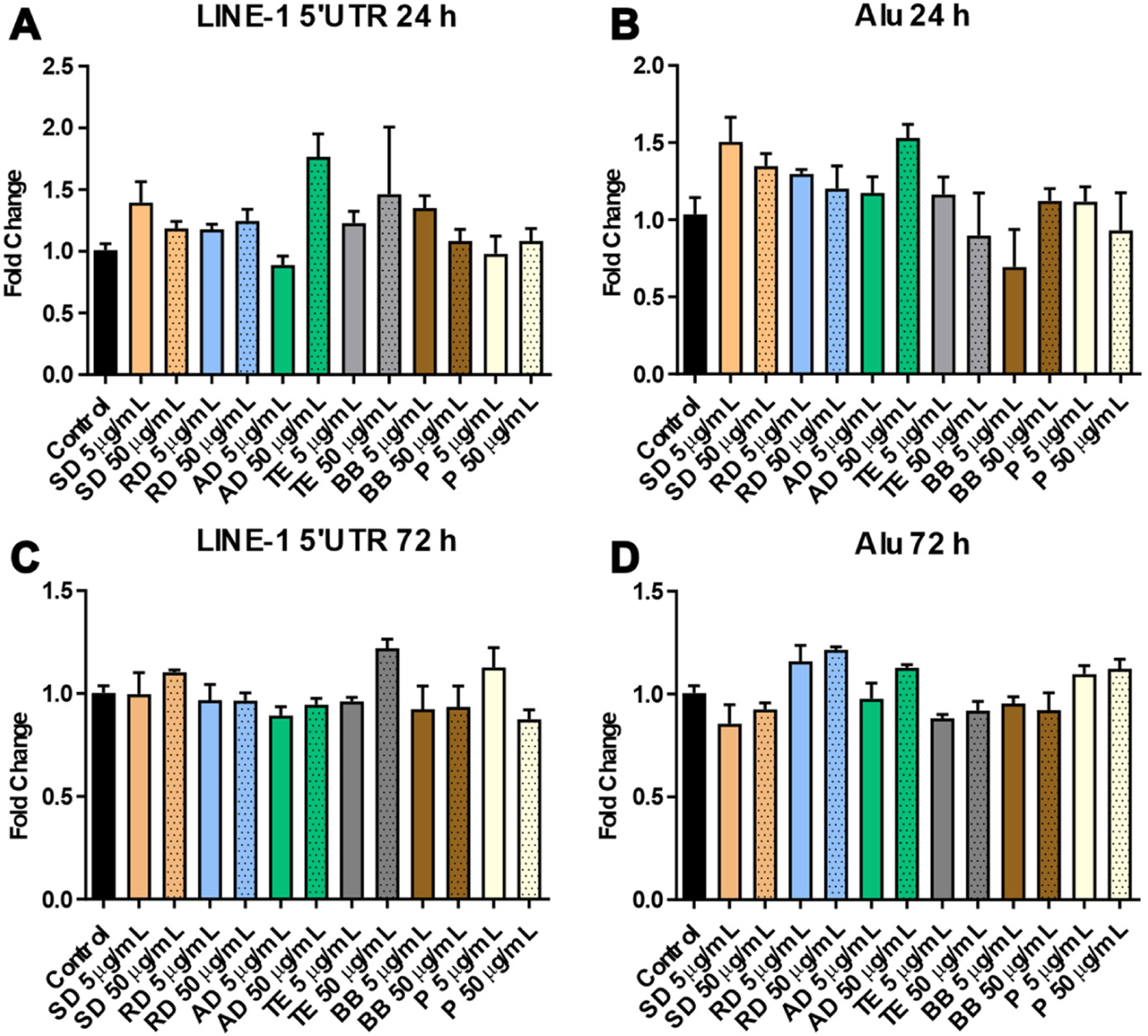
DNA methylation in LINE-1 and Alu transposable elements after 24 and 72 h. At 24 h (panels (**A**) and (**B**)) and 72 h (panels (**C**) and (**D**)) of exposure to the water-soluble fraction of six sources of particulate matter.

**Figure 5.**
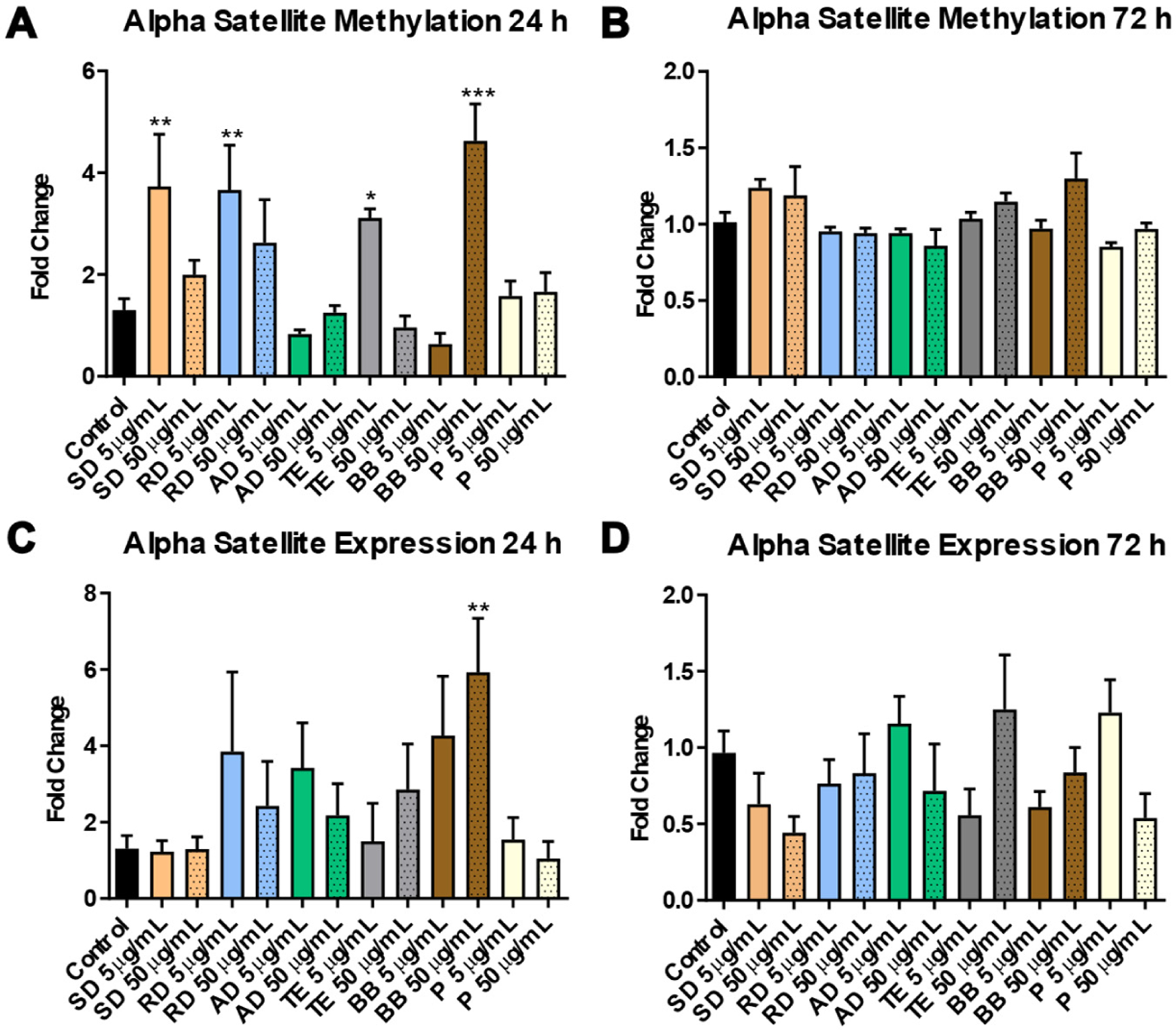
DNA methylation of alpha satellite elements at 24 h (panel (**A**)) and 72 h (panel (**C**)) and differential expression of alpha satellite elements at 24 h (panel (**B**)) and 72 h (panel (**D**)) of exposure to the water-soluble fraction of six sources of particulate matter. Asterisks: (*) denotes significant (*p* < 0.05), (**) denotes significant (*p* < 0.01), and (***) denotes significant (*p* < 0.001) difference from the control.

Analysis of two retrotransposons—LINE-1 and Alu elements—did not reveal significant changes in their DNA methylation patterns in any of the experimental groups (Figure 4). Far more pronounced alterations were observed in the DNA methylation of alpha satellites—pericentromeric repetitive elements. Interestingly enough, the observed DNA hypermethylation after exposure to SD (3.8-fold, *p* < 0.01), RD (3.7-fold, *p* < 0.05), and TE (3.1-fold, *p* < 0.05) was associated with the lower dose—5 μg/mL (Figure 5A). At the same time, an opposite effect was observed in the case of BB where only exposure to 50 μg/mL resulted in meaningful and significant DNA hypermethylation of alpha satellites (4.6-fold, *p* < 0.001). By 72 h, these effects were resolved (Figure 5B). Similarly, increased expression of alpha satellites at 24 h was observed mostly after exposure to low doses of PM (specifically in the case of RD and AD); however, these effects did not reach statistical significance, possibly due to large sample-to-sample variability. Strong overexpression was observed after exposure to both doses of BB, with exposure to 50 μg/mL reaching statistical significance (5.9-fold, *p* < 0.01) (Figure 5C). No significant changes in alpha satellite expression were observed at the 72 h time-point (Figure 5D).

### 3.2. Geospatial Analysis

**Figure 6.**
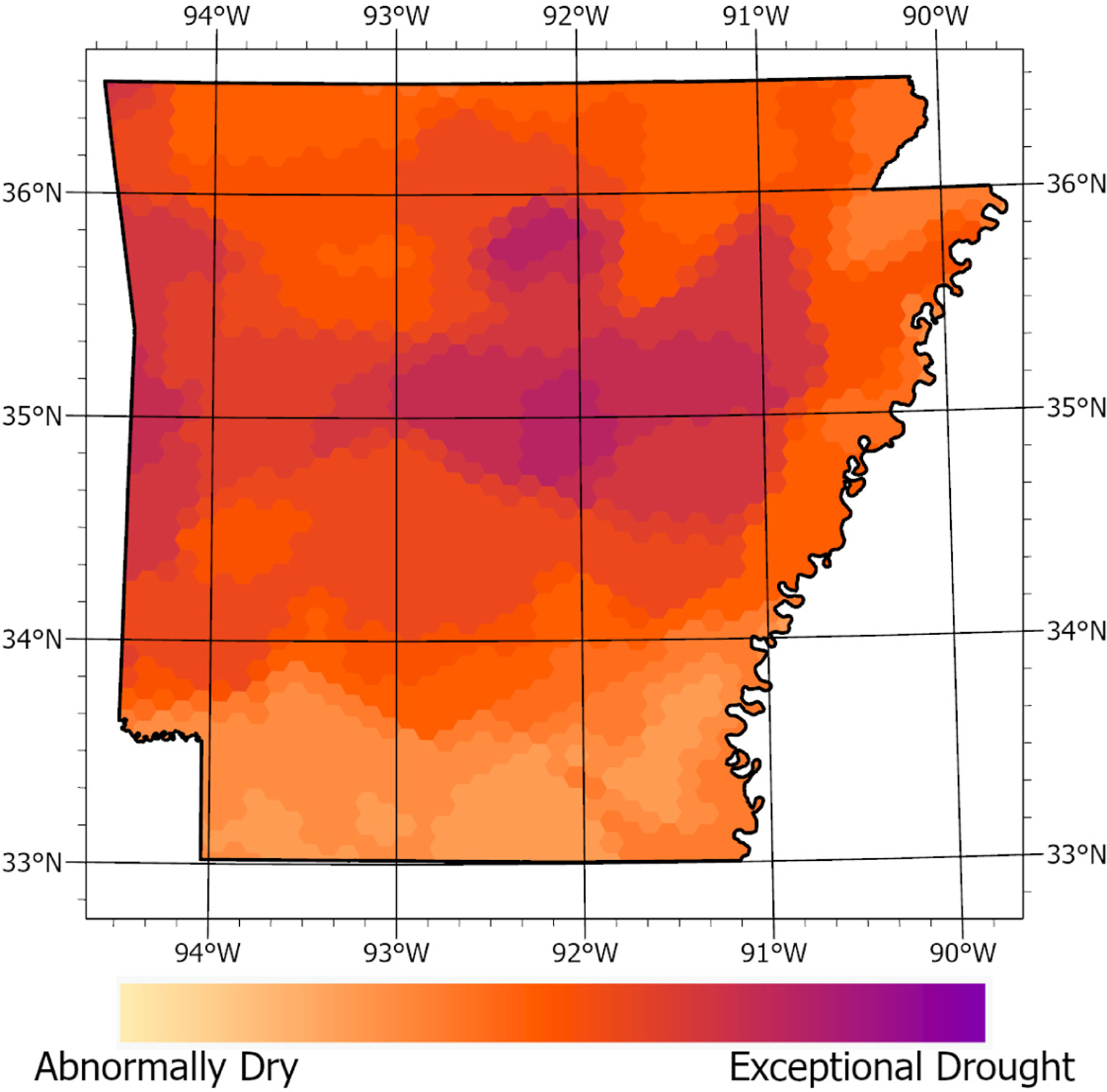
Dry conditions for the state of Arkansas from the period of 21 September 2022 through 1 November 2022.

**Figure 7.**
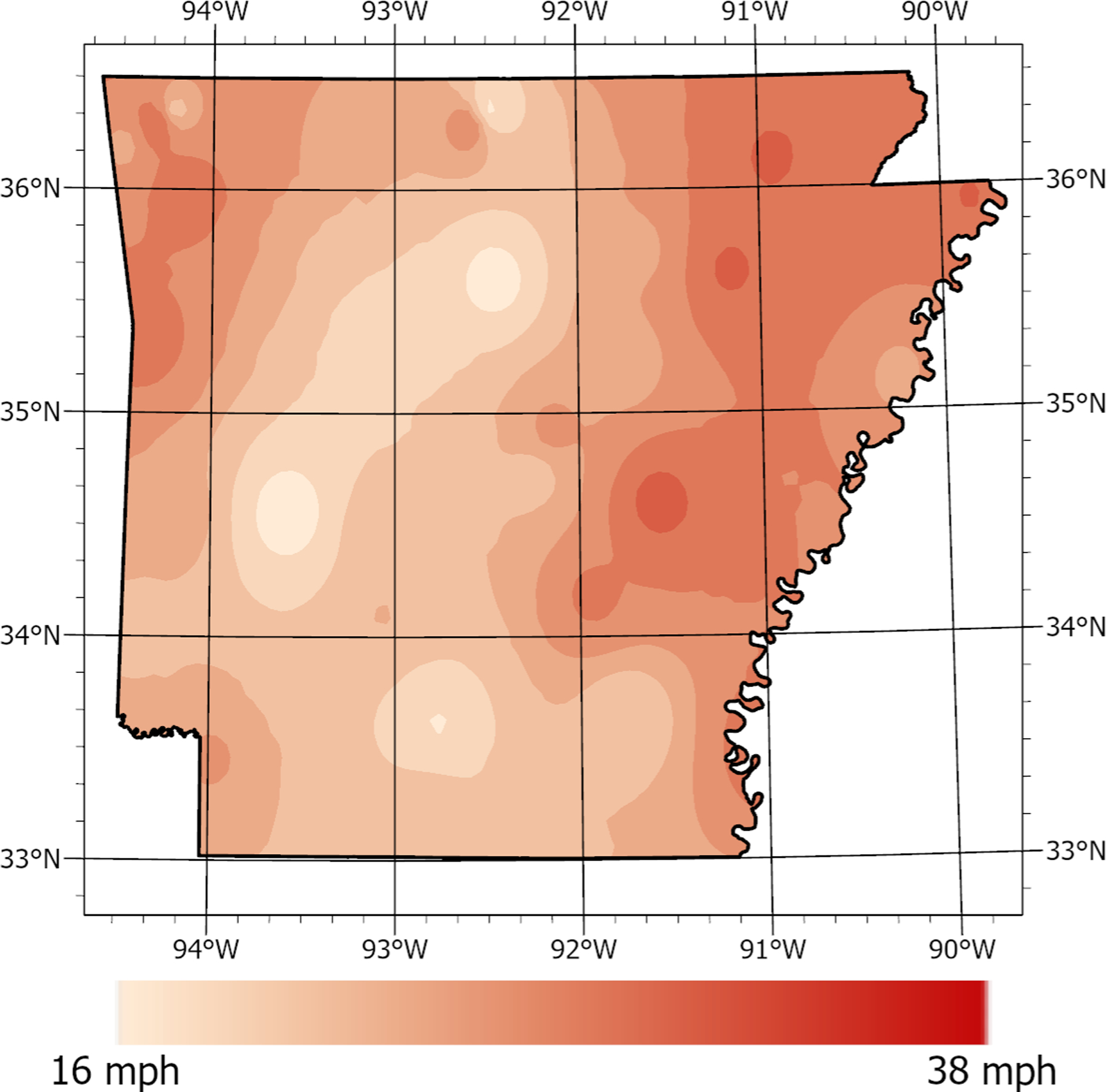
Wind speeds in the state of Arkansas.

Further geospatial analysis clearly demonstrated overwhelmingly dry conditions that the entire state of Arkansas experienced from the period of 21 September 2022 through 1 November 2022 (Figure 6). While exceptionally dry conditions were observed predominantly in central Arkansas, the Delta area—which is primarily farmland—experienced abnormally dry conditions as well. Moreover, further analysis of the wind speeds in Arkansas demonstrates the highest winds predominantly in the Delta area, with most average maximum wind speeds exceeding 16 mph (Figure 7). This is particularly important as existing models estimate that dust becomes airborne at a speed of ~6.5 m per second, which equates to 14.5 mph [31]. Thus, the agricultural dust that exhibited the most pronounced toxicological responses has a high potential to travel long distances through the unforested areas of eastern Arkansas.

## 4. Discussion

Intensifying drought is associated with an increase in wildfires, and the drift of pesticides is an emerging problem requiring significant attention. Rural communities are the most vulnerable to these effects. The results presented here indicate that, at least in the state of Arkansas, particulate matter associated with biomass burning and agricultural dust exhibits the highest degree of toxicological responses: up-regulation of markers of oxidative stress and pro-inflammatory response, as well as epigenetic alterations in human small alveolar epithelial cells.

While the presence of pro-oxidative and pro-inflammatory responses was not surprising, the extent of these responses was somewhat alarming (i.e., 29-fold up-regulation of *HMOX1* gene to 50 μg/mL dose of BB). Also, the escalation of *TNFα* mRNA levels at 72 h after initiation of exposure to AD (7-fold increase—nearly doubled from the 24 h levels) indicates strong potential for persistence of pro-inflammatory signaling associated with prolonged exposure to AD. This may have detrimental effects on farming communities and those who are exposed to AD chronically. Importantly, we demonstrated that exposure to SD—soil samples collected outside the farmlands—also caused a significant but lower extent of biological responses compared with AD. This suggests that non-natural components in the SD—likely agrochemicals—are the contributing factor to much stronger effects observed in AD compared with SD. Of particular concern in these regards are the organochlorines that not only bind strongly with soil particles but can also persist in the environment (i.e., upper layers of soil) for many years [32,33].

These findings are in agreement with the results of our previous study that investigated the effects of the same samples in RAW-264-7 macrophage cells [17]. In this experimental system, exposure to AD exhibits potent pro-inflammatory responses as well as induced high levels of cytotoxicity, induction of reactive oxygen species (ROS), and associated up-regulation of the *Hmox1* gene.

We further demonstrate an epigenetic reprogramming induced by exposure to AD and BB particles. Epigenetic alterations have been attributed to many diseases. Indeed, altered DNA methylation—one of the major epigenetic mechanisms of control over the proper expression of genetic information—has been reported in many diseases, including respiratory disease (i.e., asthma) and cancer [34,35]. Accumulating evidence indicates that epigenetic reprogramming is not only a consequence of disease but often serves as a driving mechanism in the pathogenesis of diseases [36]. Various environmental stressors—from heavy metals to ionizing radiation—are known to affect DNA methylation and DNA methylation machinery [26,37,38]. Exposure to PM, from coarse PM_10_ particles to nanoparticles has been associated with altered DNA methylation in vitro, in vivo, and in humans [39–41]. Therefore, epigenetic alterations associated with environmental exposures receive much attention as key mechanisms in the pathogenesis of exposure-associated diseases [42,43]. The first studies to recognize the role of climate change (i.e., heat) and associated epigenetic alterations in the pathogenesis of allergic and respiratory diseases have recently emerged [44].

In our study, we observed dynamic changes in the expression and enzymatic activity of DNA methyltransferases. These enzymes are responsible for either copying DNA methylation patterns to the newly synthesized strand or for methylation of the novel methylation sites. BB particles clearly demonstrated the highest potential to affect DNA methyltransferases among all tested samples. The altered status of DNA methyltransferases may result in alterations in DNA methylation, especially repetitive elements—repetitive DNA sequences that can occupy 50% or more of the entire genomes in mammalian species [45]. Exposure to environmental stressors has been associated with changes in DNA methylation of repetitive elements that, in turn, can result in their transcriptional activation and development of genomic instability [46–48]. In our study, we observed a lack of changes in the DNA methylation of LINE-1 and Alu—major repetitive elements and key retrotransposons in human genomes. This was also associated with a lack of changes in their transcriptional activity. At the same time, both DNA methylation and expression of alpha satellites were affected by exposure to several sources of PM, including SD, RD, and BB. Alpha satellites are major pericentromeric repeats whose function is centromere and kinetochore assembly and heterochromatin formation [49]. The latter is intrinsically associated with the condensed chromatin and thus the observed changes in the alpha satellite activity may be considered a defensive mechanism in response to toxic PM exposure (BB, in particular).

The results of this and our earlier study [17] and the similarities observed between them must be scrutinized from several different perspectives. Clearly, first and foremost is the public health perspective. Both BB and AD present cases of significant concern due to the ubiquitous nature of their sources; in the case of BB, it is due to extensive wildfires with the observed tendency towards increased frequency and intensity. Millions of acres a year are being directly affected by wildfires, which adversely affect human health in the state, country, and even continent. Due to the BB particles’ small size and ability to travel far, they can be detected thousands of miles away in concentrations high enough to cause acute respiratory issues or trigger exacerbation of chronic conditions, such as asthma [6]. Their small size (often <0.1 μ or PM_0.1_) allows for deeper (past alveoli) inhalation and facilitates absorption into the body, thus leading to systemic exposure.

Large-scale wildfires are usually augmented by extreme weather conditions (i.e., lightning strikes or high temperatures during dry seasons). However, it must be taken into consideration that the roots of wildfires in Arkansas (as well as some other rural states) are different. The latter often stem from arson and routine burning of trash and vegetation (both leaves in residential areas as well as remains of harvests in the farmlands) that can be further amplified by extreme weather conditions (i.e., winds, drought). Most of these factors are preventable, and appropriate communication strategies, dissemination of knowledge, and increased populational public health awareness through partnership with community organizations and key stakeholders may offer effective solutions. However, lack of resources and inadequate funding for infrastructure and education present various challenges.

Concerns associated with AD are due to the vital importance of agricultural practices for humans’ existence. Thus, millions of acres of farmlands are used in crop production and millions of pounds of agrochemicals are being introduced into the soils annually. Some of these agrochemicals or products of their degradation are characterized by a high degree of persistence in the environment. A sobering example is the case of the Aral Sea, where local communities are now forced to confront the results of the half-century-long uncontrolled usage of agrochemicals and environmentally unfriendly choices that were made in order to grow cotton in the desert. Increases in many diseases have been reported in the last several decades in communities residing within the proximity of the exposed seabed [9,10]. Similar fates are observed in the cases of several other lakes across the US [11]. In southeastern Arkansas, a number of lakes were known to be contaminated with pesticides—mostly herbicides. Two of them—Old Town Lake and Lake Chicot—are located in counties (Philips and Chico, respectively) that are known for health disparities and are characterized by extremely high rates of poverty. Both lakes were known since the 20th century to be the most contaminated lakes in the state, containing detectable levels of many agrochemicals. Concentrations of some of them, like cyanazine (a pesticide whose use in the United States was discontinued due to its toxicity), were greater than 1 μg/L (in Old Town Lake) [50]. Besides significant concerns about local fish [51] and groundwater contamination that existed for decades, recent aridification in the state threatens to substantially reduce the water levels in many reservoirs contaminated with runoff. This, together with intensified winds, may result in chronic exposures of local communities to particles contaminated with mixtures of agrochemicals, raised from the exposed lakebeds.

Similar to wildfires, the effects caused by particles entrained with agrochemical residues may be far-reaching as the latter can travel long distances, especially in areas lacking natural barriers—i.e., forests and mountains—like in southeast Arkansas. For instance, toxic dusts saturated with agrochemical residues and heavy metals were shown to be able to travel 300+ miles from the dry basin of the Aral Sea [52] and infiltrate air and food supply pathways [11].

Thus, the case of AD is even more challenging compared with BB, as it will require both mitigation and prevention strategies. Indeed, as agrochemicals were used in the state extensively for decades and levels of some—i.e., environmentally persistent organochlorines—reached significant levels, appropriate mitigation strategies will need to be developed. Challenges are expected to be similar to BB cases.

Public health issues are of utmost concern. However, it must be taken into consideration that the economic impacts that follow can be devastating as well. From the loss of the producing farmlands or resources for timber production to unemployment and decreased productivity of those that are employed (due to both external, i.e., heat and internal, i.e., poor health state—factors). Altogether, these factors will inevitably result in mass out-migration and economic collapse.

Intervention, mitigation, and prevention strategies are challenging to implement in rural communities. To produce positive change that addresses such hazards, the community must be at the forefront and be equipped with the necessary knowledge and advocacy skills to protect themselves and their communities. In addition, mitigation solutions developed in collaboration with community members are more likely to address citizens’ concerns and yield more equitable outcomes compared with policy solutions developed with no or only token participation of community members [53–57].

One way to engage with community members in building knowledge of potential hazards like this in advance of mitigation efforts is through citizen science, which is defined as the engagement of ordinary people in designing and conducting scientific research [58]. If citizen science research is applied to address the climate crisis in rural communities, it may contribute to improving community knowledge about the nature and scope of various hazards discussed in this study [59]. The success of a citizen science project depends on citizen scientists’ constant contributions and commitment to the project. Thus, it is essential to comprehend why ordinary citizens are willingly involved in this kind of community-based project from a practical standpoint. Moreover, in the literature on citizen science, this is considered one of several key research areas that need to be further explored since the extant literature appears to lack evidence-based knowledge on motivational and incentive factors to encourage lay people to participate in citizen science projects, particularly in the context of public health [60]. Another key research gap in the literature is the paucity of empirical evidence on the benefits and outcomes of a citizen science project for citizen participants. Therefore, future research on these issues can make both practical and theoretical contributions to citizen science for public health.

## 5. Conclusions

The impact of climate change on human well-being has increasingly been recognized. Aridification and wildfires are not only significant manifestations of climate change but, as evident from the results of our study, possess substantial toxicological potential and thus pose direct threats to human health. Due to socioeconomic and other factors, rural communities are among the most vulnerable populations to these effects. The insufficient knowledge on this topic and lack of resources to tackle climate change challenges dictates the necessity of the development of partnerships between scientists and rural communities. Such partnerships, in the form of citizen science projects, may provide important solutions to prevent and mitigate the negative effects of the rapidly evolving climate and improve the well-being of rural communities. Future directions should focus on further studies that will allow for a better understanding of the health effects of environmental exposures, as well as on collaborations with governmental and environmental agencies, and involvement of local communities in monitoring and addressing the identified issues.

## Data Availability

The data presented in this study are available upon request from the corresponding author.
